# 6-Year trajectory of fasting plasma glucose (FPG) and mortality risk among individuals with normal FPG at baseline: a prospective cohort study

**DOI:** 10.1186/s13098-023-01146-2

**Published:** 2023-08-13

**Authors:** Wanlu Li, Chi Pang Wen, Wenyuan Li, Zhijun Ying, Sai Pan, Yizhan Li, Zecheng Zhu, Min Yang, Huakang Tu, Yi Guo, Zhenya Song, David Ta-Wei Chu, Xifeng Wu

**Affiliations:** 1 Department of Big Data in Health Science School of Public Health, and Center of Clinical Big Data and Analytics of The Second Affiliated Hospital, Zhejiang University School of Medicine, Hangzhou, Zhejiang China; 2 National Institute for Data Science in Health and Medicine, Zhejiang University, 866 Yuhangtang Road, Hangzhou, 310058 Zhejiang China; 3 Department of Nutrition and Food Hygiene School of Public Health, Zhejiang University School of Medicine, Hangzhou, China; 4 Department of Health Management Center and Department of General Medicine, The Second Affiliated Hospital Zhejiang University School of Medicine, Hangzhou, Zhejiang China; 5 The Key Laboratory of Intelligent Preventive Medicine of Zhejiang Province, Hangzhou, Zhejiang China; 6 MJ Health Management Center, Taipei, Taiwan; 7 Cancer Center, Zhejiang University, Hangzhou, Zhejiang China; 8 School of Medicine and Health Science, George Washington University, Washington, DC USA

**Keywords:** Fasting plasma glucose, Mortality, Epidemiology, Longitudinal change, Cohort analysis, Glucose homeostasis, Group-based trajectory model

## Abstract

**Background:**

Higher fasting plasma glucose (FPG) levels were associated with an increased risk of all-cause mortality; however, the associations between long-term FPG trajectory groups and mortality were unclear, especially among individuals with a normal FPG level at the beginning. The aims of this study were to examine the associations of FPG trajectories with the risk of mortality and identify modifiable lifestyle factors related to these trajectories.

**Methods:**

We enrolled 50,919 individuals aged ≥ 20 years old, who were free of diabetes at baseline, in the prospective MJ cohort. All participants completed at least four FPG measurements within 6 years after enrollment and were followed until December 2011. FPG trajectories were identified by group-based trajectory modeling. We used Cox proportional hazards models to examine the associations of FPG trajectories with mortality, adjusting for age, sex, marital status, education level, occupation, smoking, drinking, physical activity, body mass index, baseline FPG, hypertension, dyslipidemia, cardiovascular disease or stroke, and cancer. Associations between baseline lifestyle factors and FPG trajectories were evaluated using multinomial logistic regression.

**Results:**

We identified three FPG trajectories as stable (n = 32,481), low-increasing (n = 17,164), and high-increasing (n = 1274). Compared to the stable group, both the low-increasing and high-increasing groups had higher risks of all-cause mortality (hazard ratio (HR) = 1.18 (95% CI 0.99–1.40) and 1.52 (95% CI 1.09–2.13), respectively), especially among those with hypertension. Compared to participants with 0 to 1 healthy lifestyle factor, those with 6 healthy lifestyle factors were more likely to be in the stable group (OR_low-increasing_ = 0.61, 95% CI 0.51–0.73; OR_high-increasing_ = 0.20, 95% CI 0.13–0.32).

**Conclusions:**

Individuals with longitudinally increasing FPG had a higher risk of mortality even if they had a normal FPG at baseline. Adopting healthy lifestyles may prevent individuals from transitioning into increasing trajectories.

**Supplementary Information:**

The online version contains supplementary material available at 10.1186/s13098-023-01146-2.

## Highlights

Among individuals with normal levels of fasting plasma glucose (FPG) at baseline, those who experienced upward trends in FPG over a 6-year period had a higher risk of mortality compared to individuals with relatively low and stable FPG levels.

Individuals with hypertension were more vulnerable to the adverse effects of increasing FPG trajectories on mortality.

Adopting healthy lifestyles may prevent participants with normal FPG levels at baseline from developing a higher risk of mortality.

**Supplementary Information:**

The online version contains supplementary material available at 10.1186/s13098-023-01146-2.

## Introduction

Elevated fasting plasma glucose (FPG) has been consistently associated with higher risks of all-cause and cardiovascular disease (CVD) mortality [1–6]. In China alone, the rise in type 2 diabetes (T2D)-related deaths during 2020 has contributed significantly to the burden on healthcare, society, and economy [7].

The underlying mechanisms linking high FPG levels to adverse outcomes may involve increased oxidative stress, activation of protein kinase C, and advanced glycated end product receptor activation, ultimately leading to endothelial dysfunction [8]. Such conditions are associated with a higher incidence of acute cardiovascular diseases, including myocardial infarction and stroke. Several studies have explored the associations between FPG concentrations and mortality [4, 9–15]. A recent meta-analysis of 53 prospective cohort studies [4] found that even within the range of 5.9–6.9 mmol/L, the recommended cutoff value for impaired fasting glucose, high FPG concentration was linked to a higher risk of all-cause mortality. Furthermore, long-standing diabetes has been identified as an independent risk factor contributing to mortality, particularly in the elderly population [14].

However, the potential adverse effects of higher-than-normal FPG on mortality are likely to be chronic and may develop over years. Notably, most published studies [4, 9–15] have relied on FPG measurements taken at a single time point, and therefore, the potential effects of longitudinal trends in FPG levels have not been fully explored. It remains unclear whether the increased risk of mortality is primarily attributed to the baseline FPG level or the longitudinal changes observed during the follow-up period. The failure to account for longitudinal changes in FPG could potentially bias the true association between FPG and mortality risk toward null. To clarify this association, it is crucial to conduct prospective cohort studies that assess the effects of long-term FPG trajectories on mortality. However, due to limited sample sizes and the burden of conducting multiple examinations, there is a scarcity of information on long-term FPG trajectory groups. Additionally, existing studies that have assessed longitudinal FPG levels were limited to relatively short time periods and focused exclusively on patients with T2D [16, 17].

Therefore, we aimed to estimate FPG trajectory groups over a 6-year period among 50,919 participants without diabetes at baseline in the prospective MJ Cohort study. We sought to investigate the associations of these trajectory groups with all-cause mortality and CVD mortality while assessing whether such associations differed by age, sex, body mass index (BMI), smoking status, drinking status, and hypertension. Furthermore, we evaluated the associations between baseline lifestyle factors and FPG trajectory groups.

## Research design and methods

### Study design and participants

All participants in the MJ cohort study participated in a standard health screening program organized by the Taiwan MJ Health Management Institution. Detailed information about the MJ cohort study has been reported elsewhere [18, 19]. At enrollment, each participant provided self-reported demographic information, medical history, and lifestyle details, and underwent a series of physical examinations, body measurements, blood, urine, and functional tests [20]. From 1996 to 2010, a total of 52,285 adults aged ≥ 20 years completed at least four FPG measurements with valid FPG data. Participants with diabetes at enrollment (FPG > 126 mg/dL [21], using antidiabetic medication, or self-reported T2D) were excluded (n = 1348), as well as those who were followed for less than one year (n = 18). The final study sample comprised 50,919 participants.

### Assessment of FPG and trajectory modeling

Participants were advised to fast for at least 8 h before their clinic visits. Fasting blood samples were collected in the morning and analyzed for glucose concentration (7150 autoanalyzers; Hitachi) [22].

Group-based trajectory modeling (GBTM) analysis was employed to identify distinct groups of individuals exhibiting similar patterns of change over time in FPG levels. To achieve a balance between trajectory stability and sample size, the GBTM approach [23] was applied based on at least four FPG measurements (with time intervals between each measurement less than 2.0 years) within a 6-year period after enrollment. The STATA Traj package’s censored normal model was used for this analysis.

**Fig. 1:**
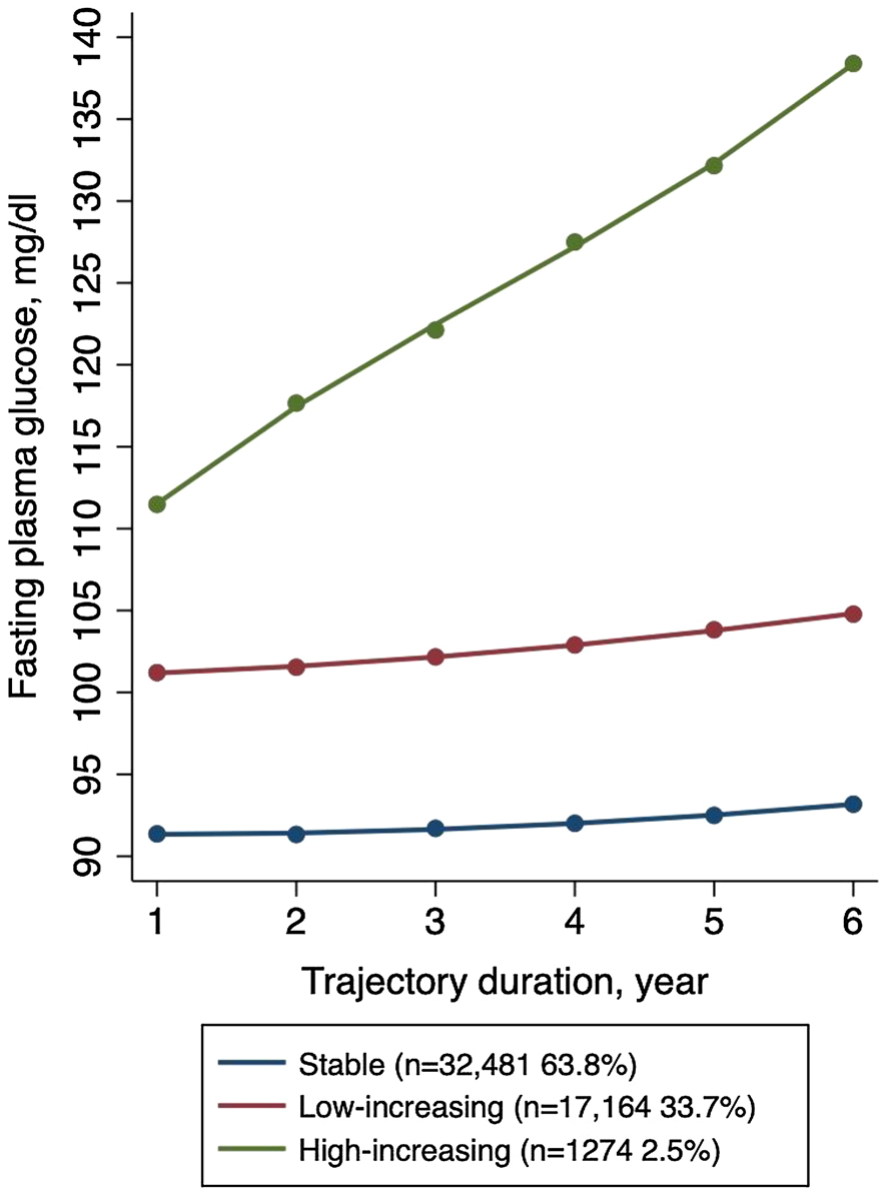
FPG trajectory groups based on at least 4 measurements within a 6-year period after baseline

The FPG level served as the dependent variable, and GBTM was repeated with 2–5 trajectory groups to determine the optimal number of groups. In order to choose the best-fitting models, three functional forms (linear, quadratic, and cubic) were considered and evaluated based on their significance levels (*P* < 0.05). The optimal trajectory shapes and number of groups were determined based on the following criteria [24, 25]: (1) the estimate of the log Bayes factor; (2) the number of participants within each trajectory group should be at least 2% of the total sample size; and (3) the average posterior probability of each trajectory group should be over 0.70. Consequently, three trajectories were identified, with two quadratic order terms and one cubic order term in the final model. The trajectory groups were named based on their visual patterns. The first trajectory group ("stable") displayed the lowest mean FPG level over the 6-year period. The second trajectory group was named "low-increasing" because it showed a moderate mean FPG level with a slight increasing trend. The last group was named "high-increasing" for similar reasons (Fig. 1). The average posterior probability for each trajectory was 0.93, 0.88, and 0.95, respectively.

### Outcome ascertainment

The study focused on three main outcomes: all-cause mortality, cardiovascular disease (CVD) mortality, and cancer mortality. Data for these outcomes were retrieved from the Taiwan Death File, a computer database containing death information coded from death certificates [19]. The mortality data were linked to the MJ health data through December 31, 2011. The underlying causes of death were identified using the International Classification of Disease (ICD), 9th (390‐459 for CVD deaths and 140‐239 for cancer deaths) and 10th Revision (I00‐I99 for CVD deaths and C00‐C97 for cancer deaths). While the recruitment of the participants was continuous from 1996 to 2010, each participant had to have at least four measurements within a 6-year period from the time of recruitment. The follow-up started from the year of the last FPG measurement until the year of death or December 31, 2011, whichever came first. The average duration of follow-up was 6.5 years (range: 1.0–12.0 years) of follow-up, and 877 deaths were recorded, including 394 cancer deaths and 151 CVD deaths.

### Covariate assessment

Covariates were selected based on subject knowledge and previous literature. The covariates included age, sex, BMI groups (underweight, normal weight, overweight, and obesity), marital status (married, unmarried, divorced, or widowed), education level (middle school or lower, high school, junior college, or college or higher), occupation (white collar, blue collar, self-employed, housewife or house husband, or other), leisure time physical activity (inactive, low, medium, high, and very high), smoking status (never, former, or current), drinking status (never, former, or current), hypertension (systolic blood pressure ≥ 140 mmHg, or diastolic blood pressure ≥ 90 mmHg, or self-reported physician-diagnosed hypertension), dyslipidemia (total cholesterol ≥ 240 mg/dL, or triglyceride ≥ 200 mg/dL or high-density lipoprotein-cholesterol < 40 mg/dL), self-reported any CVD or stroke, and self-reported any cancer.

BMI groups were defined as underweight (BMI < 18.5 kg/m^2^), normal weight (≥ 18.5 and < 24.0 kg/m^2^), overweight (≥ 24.0 and < 28.0 kg/m^2^), and obese (≥ 28.0 kg/m^2^). Leisure time physical activity was estimated by multiplying the intensity (metabolic equivalent) by duration (hours): inactive (< 3.75 MET h/week), low (≥ 3.75 and < 7.50 MET h/week), medium (≥ 7.50 and < 16.50 MET h/week), high (≥ 16.50 and < 25.50 MET h/week), and very high (≥ 25.50 MET hours/week).

### Statistical analysis

Differences between participants in each of the three FPG trajectory groups were compared using ANOVA tests for continuous variables and Chi-square tests for categorical variables.

For multivariable analysis, Cox proportional hazards models were used to estimate hazard ratios (HRs) and 95% confidence intervals (CIs) for the associations between FPG trajectory groups and mortality, with the stable trajectory group as the reference. The proportional hazard assumption was tested using Schoenfeld residuals and showed no violation with a *P*-value of 0.32. The analysis involved a two-stage modeling approach for each outcome: the first model was a crude model, while the second model adjusted for age, sex, BMI groups, marital status, education level, occupation, smoking status, drinking status, leisure time physical activity, baseline FPG, hypertension, dyslipidemia, any CVD or stroke, and cancer. Subgroup analyses were conducted, stratified by age of 50 years), sex, BMI at 24 kg/m^2^, and hypertension. Interaction tests were performed to assess the statistical significance of interactions between each potential effect modifier and trajectory groups.

Sensitivity analyses were conducted by: (1) excluding participants followed for less than three years; and (2) restricting the analysis to participants with at least 5 visits in the 6-year period, instead of 4 visits as in the main analysis. Additionally, participants were sorted based on their baseline FPG levels, grouped into three groups, where the number of individuals in each group was close to the percentage of individuals in each FPG trajectory group, and their associations with all-cause mortality risk were evaluated.

Furthermore, multinomial logistic regression models were employed to evaluate the associations of baseline lifestyle factors with FPG trajectory groups. Six baseline lifestyle factors were considered: smoking status, drinking status, physical activity (inactive, moderate, or active), BMI (< 24.0 kg/m^2^, ≥ 24.0 and < 28.0 kg/m^2^, or ≥ 28 kg/m^2^), fruits and vegetables intake, and waist circumference (WC). Healthy lifestyle factors were defined as never smoking, never drinking, active physical activity, BMI ≥ 24.0 and < 28.0 kg/m^2^, and daily consumption of both fruits and vegetables, with a WC < 90 cm for males or < 85 cm for females.

All statistical analyses were conducted using STATA version 14.0 (Stata Corporation, College Station, TX, USA), and the GBTM model was constructed using the Traj package in STATA. A two-tailed *P*-value < 0.05 was considered statistically significant.

## Results

### FPG trajectory groups

The study identified three FPG trajectory groups among the 50,919 participants. In the stable group (n = 32,481, 63.8%), mean FPG levels ranged from 5.1 to 5.2 mmol/L within the 6-year period after enrollment; in the low-increasing group (n = 17,164, 33.7%), mean FPG levels ranged from 5.6 to 5.8 mmol/L within the 6-year period, showing a slight increasing trend; and in the high-increasing group (n = 1274, 2.5%), mean FPG levels ranged from 6.2 to 7.7 mmol/L within the 6-year period, indicating a notable increasing trend.

### Baseline characteristics of study participants

Among the participants, 51.0% were male, and 49.0% were female, with a mean age of 40.1 years at the first clinic visit (Table 1). The majority were married (70.4%) and had attained a high school or above education level (81.4%). A significant proportion of participants were non-smokers (67.6%) and non-drinkers (72.3%). Leisure time physical inactivity was observed in 42.5% of the participants. The high-increasing group had a higher proportion of males, lower education level, higher BMI, higher prevalence of smoking, drinking, physical inactivity, hypertension, and dyslipidemia compared to the stable and low-increasing groups.

**Table 1:** Baseline characteristics of the study participants from the MJ cohort according to FPG trajectory groups

Characteristics	Total (N = 50,919)	FPG trajectory			*P* value
		Stable (N = 32,481)	Low-increasing (N = 17,164)	High-increasing (N = 1274)	
Age, years	40.1 [± 11.8]	37.7 [± 11.0]	43.9 [± 11.9]	50.0 [± 11.6]	< 0.001
Sex					< 0.001
Male	25,942 (51.0)	13,794 (42.5)	11,301 (65.8)	847 (66.5)	
Female	24,977 (49.0)	18,687 (57.5)	5863 (34.2)	427 (33.5)	
Marital status					< 0.001
Unmarried	10,543 (20.7)	8182 (25.2)	2290 (13.3)	71 (5.6)	
Married	35,820 (70.4)	21,681 (66.8)	13,116 (76.4)	1023 (80.3)	
Divorced or widowed	2595 (5.1)	1442 (4.4)	1041 (6.1)	112 (8.8)	
Education level					< 0.001
Middle school or lower	9471 (18.6)	4999 (15.4)	4028 (23.5)	444 (34.9)	
High school	11,302 (22.2)	7400 (22.8)	3626 (21.1)	276 (21.7)	
Junior college	12,131 (23.8)	8325 (25.6)	3619 (21.1)	187 (14.7)	
College or higher	16,612 (32.6)	10,895 (33.5)	5402 (31.5)	315 (24.7)	
Occupation					< 0.001
White collar	24,848 (48.8)	16,026 (49.3)	8265 (48.2)	557 (43.7)	
Blue collar	7963 (15.6)	4859 (15.0)	2925 (17.0)	179 (14.1)	
Self employed	3630 (7.1)	2309 (7.1)	1214 (7.1)	107 (8.4)	
House wife/husband	6996 (13.7)	4336 (13.4)	2414 (14.1)	246 (19.3)	
Other	5328 (10.5)	3636 (11.2)	1580 (9.2)	112 (8.8)	
Body mass index, kg/m^2^	22.9 [± 3.3]	22.1 [± 3.1]	24.1 [± 3.2]	25.9 [± 3.4]	< 0.001
Smoking status					< 0.001
Never	34,440 (67.6)	22,839 (70.3)	10,845 (63.2)	756 (59.3)	
Former	3226 (6.3)	1648 (5.1)	1460 (8.5)	118 (9.3)	
Current	9921 (19.5)	5803 (17.8)	3812 (22.2)	306 (24.0)	
Drinking status					< 0.001
Never	36,793 (72.3)	24,451 (75.3)	11,551 (67.3)	791 (62.1)	
Former	1103 (2.2)	589 (1.8)	464 (2.7)	50 (3.9)	
Current	9483 (18.6)	5155 (15.9)	3992 (23.3)	336 (26.4)	
Physical activity					< 0.001
Inactive	21,657 (42.5)	14,527 (44.7)	6652 (38.8)	478 (37.5)	
Low active	13,518 (26.6)	8845 (27.2)	4380 (25.5)	293 (23.0)	
Medium active	9225 (18.1)	5468 (16.8)	3467 (20.2)	290 (22.8)	
High active	2812 (5.5)	1531 (4.7)	1193 (7.0)	88 (6.9)	
Very high active	1559 (3.1)	822 (2.5)	689 (4.0)	48 (3.8)	
Hypertension					< 0.001
No hypertension	27,014 (53.1)	19,899 (61.3)	6791 (39.6)	324 (25.4)	
Pre-hypertension	15,405 (30.3)	8868 (27.3)	6115 (35.6)	422 (33.1)	
Hypertension	8500 (16.7)	3714 (11.4)	4258 (24.8)	528 (41.4)	
Dyslipidemia	16,518 (32.4)	8691 (26.8)	7129 (41.5)	698 (54.8)	< 0.001
FPG, mmol/L	5.3 [0.5]	5.1 [0.3]	5.6 [0.4]	6.2 [0.5]	< 0.001

Values are presented as N (%) or Mean [± SD]

Missing data not shown

### FPG trajectory groups with all-cause and CVD mortality

Compared to the stable group, both the low-increasing and high-increasing groups showed significantly higher risks of all-cause mortality. After adjustment for age, sex, BMI, marital status, education level, baseline FPG, occupation, leisure time physical activity, smoking status, drinking status, hypertension, dyslipidemia, CVD or stroke, and cancer, the HRs for the low-increasing and high-increasing groups were 1.18 (95% CI 0.99–1.41) and 1.52 (95% CI 1.09–2.13), respectively. Similarly, both the low-increasing (HR = 1.28, 95% CI 0.85–1.95) and high-increasing groups (HR = 2.13, 95% CI 1.00–4.54) had higher risks of CVD mortality (Table 2) compared to the stable group.

**Table 2:** The risk of all-cause mortality, CVD mortality, and cancer mortality, according to FPG trajectory groups and baseline FPG groups

	Cases	Total	Model 1 HR (95% CI)	Model 2 HR (95% CI)
FBG trajectory groups				
All-cause mortality				
Stable	419	32,481	Reference	Reference
Low-increasing	397	17,164	2.02 (1.76, 2.32)***	1.18 (0.99, 1.41)
High-increasing	61	1274	3.96 (3.03, 5.18)***	1.52 (1.09, 2.13)*
CVD mortality				
Stable	68	32,481	Reference	Reference
Low-increasing	70	17,164	2.19 (1.57, 3.06)***	1.28 (0.85, 1.95)
High-increasing	13	1274	5.18 (2.86, 9.37)***	2.13 (1.00, 4.54)*
Cancer mortality				
Stable	201	32,228	Reference	Reference
Low-increasing	169	16,950	1.80 (1.47, 2.21)***	1.01 (0.78, 1.31)
High-increasing	24	1260	3.28 (2.15, 5.00)***	1.10 (0.65, 1.86)
Baseline FPG group				
All-cause mortality^a^				
FPG < 5.44 mmol/L	455	32,664	Reference	Reference
FPG ≥ 5.44 and < 6.33 mmol/L	371	16,931	1.71 (1.49, 1.96)***	0.99 (0.78, 1.25)
FPG ≥ 6.33 mmol/L	51	1,324	3.02 (2.26, 4.04)***	1.02 (0.61, 1.69)
Excluding individuals followed < 3 years				
All-cause mortality				
Stable	346	26,943	Reference	Reference
Low-increasing	321	13,357	2.02 (1.74, 2.35)***	1.14 (0.94, 1.38)
High-increasing	53	992	4.21 (3.15, 5.62)***	1.50 (1.04, 2.17)*
Using five FPG examinations in a 6-year period after baseline				
All-cause mortality				
Stable	161	14,034	Reference	Reference
Low-increasing	200	9550	2.02 (1.64, 2.49)***	1.44 (1.12, 1.86)**
High-increasing	43	1048	3.96 (2.83, 5.54)***	1.91 (1.22, 2.98)**

Model 1: crude model

Model 2: adjusted for age, sex, BMI, marital status, education level, smoking status, drinking status, physical activity, baseline FPG, hypertension, dyslipidemia, self-reported cancer at baseline, self-reported cardiovascular disease at baseline. Significance levels: *P < 0.05, **P < 0.01, ***P < 0.001

a: the number of individuals in each FPG group corresponds to the percentage of individuals in each FPG trajectory group

### Stratified analysis and sensitivity analyses

Among participants with hypertension, only the high-increasing group showed a higher risk of all-cause mortality compared to the stable group. No significant differences were observed in the longitudinal associations between FPG trajectory groups and all-cause mortality among age groups, sex, or individuals with a BMI of 24.0 kg/m^2^ (Additional file 1: Fig. S1).

Excluding participants who were followed for less than 3 years and restricting analyses to participants with at least five visits within the 6-year period yielded consistent results (Table 2). Baseline FPG groups were not associated with all-cause mortality (Table 2).

### Lifestyle factors and FPG trajectory groups

Multinomial logistic regression models revealed several associations between baseline lifestyle factors and FPG trajectory groups. Current drinkers had higher odds of belonging to the low-increasing (OR = 1.18, 95% CI 1.08–1.28) and high-increasing groups (OR = 1.25, 95% CI 1.01–1.54) compared to never drinkers. Overweight and obese participants were more likely to be in the low-increasing (OR = 1.54, 95% CI 1.44–1.66 for overweight and OR = 1.93, 95% CI 1.66–2.24 for obese, respectively) and high-increasing groups (OR = 2.49, 95% CI 2.04–3.04 for overweight and OR = 4.83, 95% CI 3.54–6.59 for obese, respectively) compared to those with normal BMI. Abdominal obesity was associated with a higher odds of being in the high-increasing group (OR = 1.27, 95% CI 1.01–1.60). Physically active individuals had lower odds of being in the low-increasing (OR = 0.93, 95% CI 0.86–1.00) and high-increasing groups (OR = 0.79, 95% CI 0.65–0.97) compared to those with inactive physical activity (Table 3).

**Table 3:** Associations of lifestyle factors and FPG trajectory groups

Lifestyle factors	Low-increasing vs stable	High-increasing vs stable
	OR (95% CI)	OR (95% CI)
Smoking status		
Never	Reference	Reference
Former	0.99 (0.87, 1.12)	1.05 (0.78, 1.43)
Current	0.96 (0.88, 1.04)	1.23 (0.99, 1.53)
Drinking status		
Never	Reference	Reference
Former	1.13 (0.93, 1.39)	1.02 (0.65, 1.63)
Current	1.18 (1.08, 1.28)***	1.25 (1.01, 1.54)*
Physical activity		
Inactive	Reference	Reference
Moderate	0.97 (0.90, 1.04)	0.91 (0.73, 1.13)
Active	0.93 (0.86, 1.00)*	0.79 (0.65, 0.97)*
Body mass index, kg/m^2^		
< 24	Reference	Reference
24–28	1.54 (1.44, 1.66)***	2.49 (2.04, 3.04)***
≥ 28	1.93 (1.66, 2.24)***	4.83 (3.54, 6.59)***
Dietary habits		
Non-daily eating fruits and vegetables	Reference	Reference
Eating both fruits and vegetables daily	1.00 (0.94, 1.06)	0.96 (0.81, 1.13)
Waist circumference (WC)		
WC < 90 cm (male)/85 cm (female)	Reference	Reference
WC ≥ 90 cm (male)/85 cm (female)	1.01 (0.90, 1.13)	1.27 (1.01, 1.60)*
Number of healthy lifestyle factors		
0–1	Reference	Reference
2–3	0.80 (0.67, 0.95)**	0.66 (0.47, 0.92)*
4	0.74 (0.63, 0.89)***	0.48 (0.34, 0.68)***
5	0.64 (0.53, 0.76)***	0.30 (0.20, 0.43)***
6	0.61 (0.51, 0.73)***	0.20 (0.13, 0.32)***

Adjusted for age, sex, marital status, education level, baseline FPG levels, hypertension, dyslipidemia, self-reported cancer at baseline, self-reported cardiovascular disease at baseline

Significance levels: *P < 0.05, **P < 0.01, ***P < 0.001

Furthermore, the odds of being in the low-increasing and high-increasing groups decreased progressively with the increase in the number of healthy lifestyle factors. Participants with six healthy lifestyle factors were more likely to be in the stable group (OR = 0.61, 95% CI 0.51–0.73 and OR = 0.20, 95% CI 0.13–0.32) compared to those with 0 to 1 healthy lifestyle factor (Table 3).

## Discussion

In this large prospective cohort study, we successfully identified three distinct FPG trajectory groups over a 6-year period and found that both low-increasing and high-increasing groups were consistently associated with a higher risk of mortality compared to the stable group. We further identified lifestyle factors that were associated with being in low- and high-increasing groups, providing supportive evidence for targeted intervention. To our knowledge, this is among the first studies that reported the associations between trajectories of long-term FPG and mortality risk among Asian populations.

Previous studies have been generally based on single-point FPG measurements and reported associations between high FPG levels and higher risk of mortality. A meta-analysis, including 97 prospective studies, found significant associations between FPG levels greater than 100 mg per deciliter and the risk of death [2]. However, FPG levels are influenced by multiple factors and change over time, making single measurements susceptible to measurement error and inadequate in reflecting glucose homeostasis [17]. Recognizing that the adverse effects of FPG on mortality are chronic, it becomes essential to capture the dynamic change in FPG over the years. Thus, models incorporating FPG trajectory data may have higher predictive power for mortality than those based on a single measurement [25]. In this study, we estimated the long-term trajectories in FPG based on at least 4 measurements within 6 years. Few cohort studies have performed such analysis due to several reasons, including limited sample sizes or the burden of collecting multiple examinations. To date, we found only 2 studies that have assessed long-term FPG trajectories and mortality [16, 17]. One study, including 6400 participants aged ≥ 30 years with T2D, showed that the trajectory of fluctuation (elevated and decreasing) was associated with an increased risk of all-cause mortality compared with the normal trajectory (HR = 1.81, 95% CI = 1.38–2.38) [17]. Another study in patients with T2D reported that an increasing trajectory of FPG is associated with a 2.1- and 3.3-fold increase in all-cause mortality and CVD mortality, respectively [16]. Our study revealed that participants in the high-increasing group had a 1.5- and 2.1-fold higher all-cause mortality and CVD mortality, respectively. The slight discrepancy may be due to the characteristics of our study population: participants with T2D at baseline were excluded from our study, while the prior studies enrolled T2D patients.

Several underlying biological mechanisms may be involved in the associations between FPG trajectory groups and mortality. Abnormal glucose status may induce oxidative stress, impairing the relaxation of smooth muscle cells and damaging the endothelial function [8, 26]. It may activate adhesion molecules and thicken the intima by excessive production of advanced glycosylation end-products and cytokines [10], contributing to diabetic angiopathy inflammation and cellular damage [27]. Moreover, unstable FPG levels might indicate other preclinical conditions, such as poor dietary intake and noncompliance with therapies [3], resulting in an increase in mortality [3, 10].

Some previous studies have demonstrated that adopting healthy lifestyles, such as engaging in active physical activity, maintaining a normal BMI, and following a healthy dietary habit, could improve glucose homeostasis [28–31]. Likewise, we found that participants who maintained active physical activity were more likely to belong to the stable group, and this likelihood increased with the number of healthy lifestyle factors.

We further examined whether the associations between longitudinal changes in FPG levels and mortality differed by individual characteristics. Interestingly, our study indicated that the high-increasing group showed a higher risk of all-cause mortality. Interestingly, our study indicated that the high-increasing group showed a higher all-cause mortality risk among participants with hypertension. This observation could be explained by the increased susceptibility of individuals with T2D to the adverse health effects of hypertension on macrovascular and microvascular complications [32], leading to elevated mortality risk. Different from our findings, some previous studies reported that FPG levels were more strongly associated with mortality risk among participants who were older, male, or had a higher BMI [1, 33, 34]. The inconsistencies may be attributed to the distinct characteristics of different study populations.

The current study had several potential limitations. First, we cannot establish causality between FPG trajectory groups and mortality due to the subjective nature of trajectory models, as they were determined based on statistics and criteria determined by the researchers. Second, because our study participants were those who voluntarily entered a comprehensive medical screening program offered by a private organization, most of them were generally healthy with better socioeconomic status. Thus, the trajectory groups identified in the current study may not be generalizable to populations with different ethnicities or age groups.

Despite these limitations, our study boasts several strengths. First, we conducted the current study in a relatively large cohort and followed for an extended period, enabling data collection on a wide range of potential confounders to decrease potential confounding. Second, we identified the FPG trajectory groups based on data collected within a 6-year period, which was more representative of the long-term trend of FPG change. This approach utilized detailed information on the changes in FPG levels during the follow-up, which could not be achieved through the traditional method using FPG levels measured at 2 time points.

## Conclusion

We identified three distinct FPG trajectory groups and revealed that both the low-increasing and high-increasing groups were associated with a higher risk of mortality in individuals with FPG levels within the normal range at baseline. These findings have important clinical implications, suggesting that individuals with normal FPG levels but unhealthy lifestyles should closely monitor their future FPG trajectories. Furthermore, the importance of maintaining low and stable FPG levels over time, particularly for individuals with hypertension, was underscored by our results. However, further investigations with long-term repeated assessments of FPG are required to validate our findings, especially in diverse ethnic populations. Incorporating neural network analysis in future studies, if feasible, may also provide a prediction model that could offer valuable insights into individual risk profiles and aid in targeted interventions for improved health outcomes.

### Supplementary Information

**Additional file 1: Figure S1.** Multivariable-adjusted association (Model 2) between high-increasing FPG trajectory groups and all-cause mortality by age (A), sex (B), hypertension (C) and BMI (D).

## Data Availability

Original datasets from MJ cohort are available to researchers. Access to data can be requested from http://www.mjhrf.org/.

## References

[1] Lee G, Kim SM, Choi S (2018). The effect of change in fasting glucose on the risk of myocardial infarction, stroke, and all-cause mortality: a nationwide cohort study. Cardiovasc Diabetol.

[2] Rao K, Seshasai S, Kaptoge S, Thompson A (2011). Diabetes mellitus, fasting glucose, and risk of cause-specific death. New Engl J Med.

[3] Xu H, Zhang Y, Xu W (2021). Associations of visit-to-visit fasting glucose with risk of mortality: a retrospective cohort study of 48,077 people with type 2 diabetes. Diabetes Metab.

[4] Cai X, Zhang Y, Li M (2020). Association between prediabetes and risk of all cause mortality and cardiovascular disease: updated meta-analysis. BMJ.

[5] Kim YS, Park YM, Han KD (2021). Fasting glucose level and all-cause or cause-specific mortality in Korean adults: a nationwide cohort study. Korean J Intern Med.

[6] De Abreu LLF, Holloway KL, Mohebbi M (2017). All-cause mortality risk in australian women with impaired fasting glucose and diabetes. J Diabetes Res.

[7] Wang F, Wang W, Yin P (2022). Mortality and years of life lost in diabetes mellitus and its subcategories in China and its provinces, 2005–2020. J Diab Res.

[8] Khatri JJ, Johnson C, Magid R (2004). Vascular oxidant stress enhances progression and angiogenesis of experimental atheroma. Circulation.

[9] Charoensri S, Kritmetapak K, Tangpattanasiri T (2021). The impact of new-onset diabetes mellitus and hypertension on all-cause mortality in an apparently healthy population: a ten-year follow-up study. J Diabetes Res.

[10] Kim SM, Lee G, Choi S (2020). Association of early-onset diabetes, prediabetes and early glycaemic recovery with the risk of all-cause and cardiovascular mortality. Diabetologia.

[11] Liu L, Chen X, Liu Y (2019). The association between fasting plasma glucose and all-cause and cause-specific mortality by gender: the rural Chinese cohort study. Diabetes Metab Res Rev.

[12] Yi SW, Park S, Lee YH (2018). Fasting glucose and all-cause mortality by age in diabetes: A prospective cohort study. Diabetes Care.

[13] Huang Y, Cai X, Mai W (2016). Association between prediabetes and risk of cardiovascular disease and all cause mortality: systematic review and meta-analysis. BMJ.

[14] Tang O, Matsushita K, Coresh J (2020). Mortality implications of prediabetes and diabetes in older adults. Diabetes Care.

[15] Rhee EJ, Jung I, Kwon H (2020). Increased mortality burden in young Asian subjects with dysglycemia and comorbidities. J Clin Med.

[16] Lee CL, Sheu WHH, Lee IT (2018). Trajectories of fasting plasma glucose variability and mortality in type 2 diabetes. Diabetes Metab.

[17] Lin CC, Li CI, Liu CS (2021). Three-year trajectories of metabolic risk factors predict subsequent long-term mortality in patients with type 2 diabetes. Diabetes Res Clin Pract.

[18] Wu X, Tsai SP, Tsao CK (2017). Cohort profile: The Taiwan MJ Cohort: half a million Chinese with repeated health surveillance data. Int J Epidemiol.

[19] Wen CP, Cheng TYD, Tsai MK (2008). All-cause mortality attributable to chronic kidney disease: a prospective cohort study based on 462293 adults in Taiwan. The Lancet.

[20] Tu H, Wen CP, Tsai SP (2018). Cancer risk associated with chronic diseases and disease markers: prospective cohort study. BMJ.

[21] The American Diabetes Association. Classification and diagnosis of diabetes: standards of medical care in diabetes-2021. Diabetes Care, 2020, 44(Supplement_1): S15-S33. 10.2337/dc21-S002 33298413

[22] Sun F, Tao Q, Zhan S (2010). Metabolic syndrome and the development of chronic kidney disease among 118924 non-diabetic Taiwanese in a retrospective cohort. Nephrology.

[23] Nagin DS (1999). Analyzing developmental trajectories: a semiparametric, group-based approach. Psychol Methods.

[24] Nagin DS (2014). Group-based trajectory modeling: an overview. Ann Nutr Metab.

[25] Yuan Z, Yang Y, Wang C (2018). Trajectories of long-term normal fasting plasma glucose and risk of coronary heart disease: a prospective cohort study. J Am Heart Assoc.

[26] Piconi L, Quagliaro L, Da Ros R (2004). Intermittent high glucose enhances ICAM-1, VCAM-1, E-selectin and interleukin-6 expression in human umbilical endothelial cells in culture: the role of poly (ADP-ribose) polymerase. J Thromb Haemost.

[27] Yim S, Malhotra A, Veves A (2007). Antioxidants and CVD in diabetes: Where do we stand now?. Curr DiabRep.

[28] Lu L, Chen Y, Cai Y (2021). Physical activity and fasting glucose in adults with abnormal glucose metabolism: findings from two independent cross-sectional studies in China. Obes Res Clin Pract.

[29] Jiang Q, Li JT, Sun P (2022). Effects of lifestyle interventions on glucose regulation and diabetes risk in adults with impaired glucose tolerance or prediabetes: a meta-analysis. Arch Endocrinol Metab.

[30] Sallar A, Dagogo-Jack S (2020). Regression from prediabetes to normal glucose regulation: State of the science. Exp Biol Med.

[31] Wang Y, Dzubur E, James R (2022). Association of physical activity on blood glucose in individuals with type 2 diabetes. Translat Behav Med.

[32] Perreault L, Pan Q, Aroda VR (2017). Exploring residual risk for diabetes and microvascular disease in the Diabetes Prevention Program Outcomes Study (DPPOS). Diabet Med.

[33] Wu M, Lu J, Yang Z (2021). Longitudinal changes in fasting plasma glucose are associated with risk of cancer mortality: a Chinese cohort study. Cancer Med.

[34] Jung MH, Yi SW, An SJ (2020). Complex interaction of fasting glucose, body mass index, age and sex on all-cause mortality: a cohort study in 15 million Korean adults. Diabetologia.

