# Supplementary material for: 6-Year trajectory of fasting plasma glucose (FPG) and mortality risk among individuals with normal FPG at baseline: a prospective cohort study
Source: Diabetol Metab Syndr. 2023 Aug 13;15:169. doi: 10.1186/s13098-023-01146-2 (PMC10424387; doi:10.1186/s13098-023-01146-2)
Supplement: Supplementary file 1 — Additional file 1: Figure S1. Multivariable-adjusted association (Model 2) between high-increasing FPG trajectory groups and all-cause mortality by age (A), sex (B), hypertension (C) and BMI (D). [file 13098_2023_1146_MOESM1_ESM.pdf]

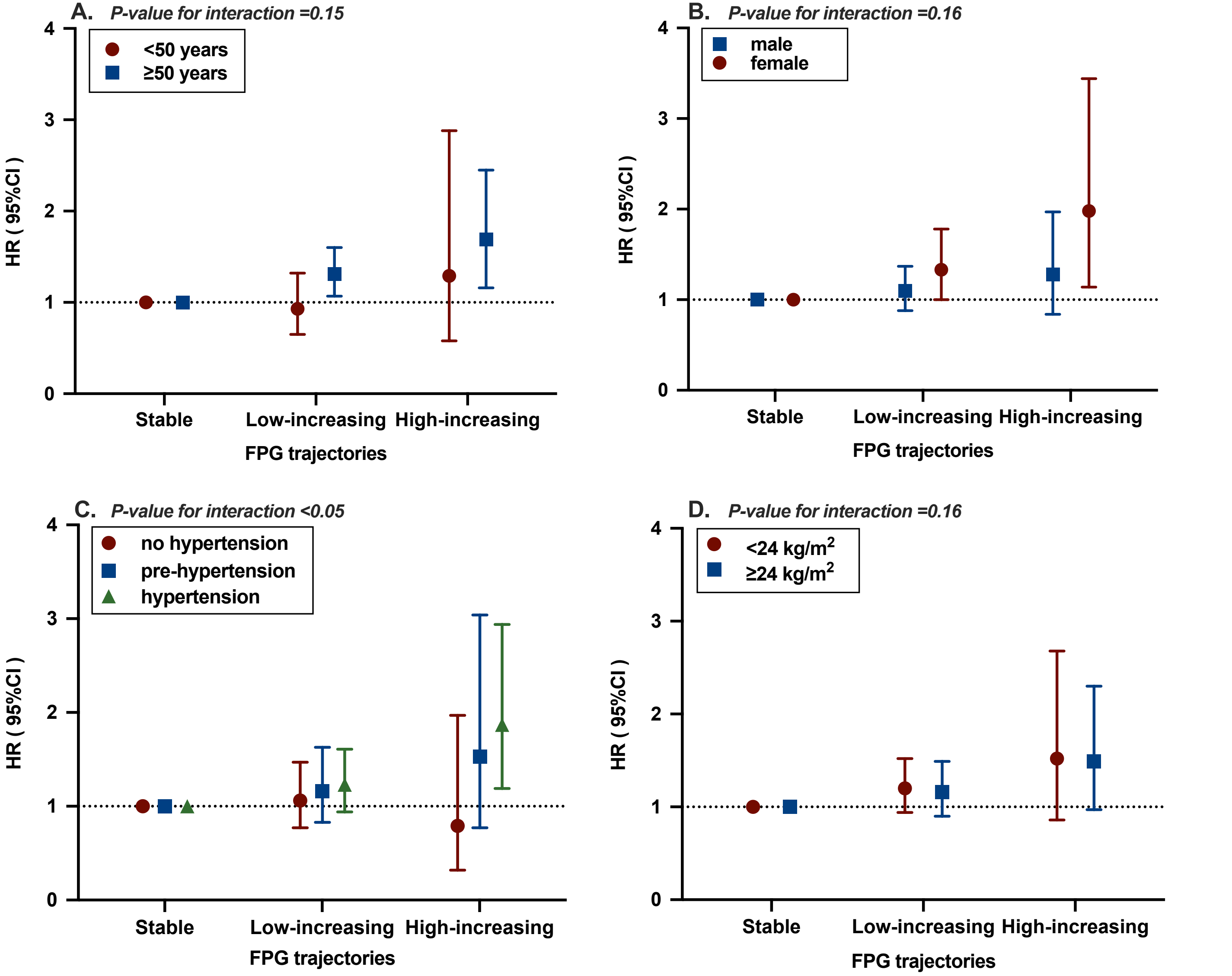

Supplementary figure 1. Multivariable-adjusted association (Model 2) between high-increasing FPG trajectory groups and all-cause mortality by age (A), sex (B), hypertension (C) and BMI (D). Model 2 was adjusted for age, sex, BMI, marital status, education level, smoking status, drinking status, physical activity, baseline FPG, hypertension, dyslipidemia, self-reported cancer at baseline, self-reported cardiovascular disease at baseline.
